# Whole‐body vibration for patients with nonalcoholic fatty liver disease: a 6‐month prospective study

**DOI:** 10.14814/phy2.14062

**Published:** 2019-05-13

**Authors:** Sechang Oh, Natsumi Oshida, Noriko Someya, Tsuyoshi Maruyama, Tomonori Isobe, Yoshikazu Okamoto, Taeho Kim, Bokun Kim, Junichi Shoda

**Affiliations:** ^1^ The Center of Sports Medicine and Health Sciences Tsukuba University Hospital Tsukuba Ibaraki Japan; ^2^ Faculty of Medicine University of Tsukuba Tsukuba Ibaraki Japan; ^3^ Graduate School of Comprehensive Human Sciences University of Tsukuba Tsukuba Ibaraki Japan; ^4^ Department of Rehabilitation University of Tsukuba Hospital Tsukuba Ibaraki Japan; ^5^ Department of Diagnostic Radiology University of Tsukuba Hospital Tsukuba Ibaraki Japan; ^6^ Faculty of Sports Health Care Inje University Gimhae Gyeongsangnamdo Republic of Korea

**Keywords:** Exercise, liver function tests, liver steatosis, liver stiffness, whole‐body vibration

## Abstract

Physical exercise has demonstrated benefits for managing nonalcoholic fatty liver disease (NAFLD). However, in daily life maintaining exercise without help may be difficult. A whole‐body vibration device (WBV) has been recently introduced as an exercise modality that may be suitable for patients who have difficulty engaging in exercise. We tested WBV in patients with NAFLD and estimated its effectiveness. We studied the effects of a 6‐month WBV program on hepatic steatosis and its underlying pathophysiology in 25 patients with NAFLD. Seventeen patients with NAFLD were designated as a control group. After WBV exercise, body weight in the study group decreased by only 2.5% compared with the control group. However, we found significant increases in muscle area (+2.6%) and strength (+20.5%) and decreases in fat mass (−6.8%). The hepatic (−9.9%) and visceral (−6.2%) fat content also significantly decreased (*P *< 0.05). There was substantial lowering of hepatic stiffness (−15.7%), along with improvements in the levels of inflammatory markers; tumor necrosis factor alpha (−50.9%), adiponectin (+12.0%), ferritin (−33.2%), and high‐sensitivity C‐reactive protein (−43.0%) (*P *< 0.05). These results suggest that WBV is an exercise option for patients with NAFLD that is effective, efficient, and convenient.

## Introduction

Nonalcoholic fatty liver disease (NAFLD) is a highly prevalent disease that presents on a spectrum ranging from asymptomatic simple steatosis to nonalcoholic steatohepatitis (NASH). It often advances to fibrosis and cirrhosis (Angulo 2002). NAFLD is the most common cause of abnormal liver function observed in clinical practice, and is a well‐described cause of metabolic abnormalities (Angulo 2002; Targher et al. 2006). Nevertheless, treatment options for NAFLD are limited. Until recently, there has been a paucity of evidence in favor of pharmacological and surgical treatments for NAFLD (Sasaki et al. 2014; Hardy et al. 2015). Many such approaches are too expensive and risky to be attempted in NAFLD patients. However, lifestyle management by diet and/or exercise aimed at weight loss has been demonstrated to be effective as a first‐line therapy (Harrison and Day 2007; Musso et al. 2012).

The specific effects of exercise have received a great deal of attention. Our previous studies (Oh et al. 2013, 2017) and those of others (Keating et al. 2015a) have clearly demonstrated the therapeutic effects of exercise regardless of detectable reductions in body weight. In addition, epidemiological studies have shown that higher levels of physical activity are associated with a lower risk of NAFLD (Rector and Thyfault 2011), and have also shown an inverse correlation between risk factors for NAFLD and cardiorespiratory fitness (Spassiani and Kuk 2008). It would appear obvious that exercise regimens are indispensable for managing NAFLD.

In the absence of specific exercise guidelines for NAFLD, various modalities and intensities of exercise have been explored (including aerobic‐type jogging, walking and cycling, resistance‐type exercise using dumbbells and barbells, and weight machines) (Keating et al. 2015a; Oh et al. 2014, 2015a,b). Studies involving these modalities have demonstrated some success. However, in daily life, maintaining these exercise routines without expert help may be difficult, due to insufficient time, risk of injury, boredom, and fatigue. Especially for obese and/or older patients with NAFLD, care must be taken with regard to mechanical factors, including hip and knee joints, as well as physiological factors including regulation of cardiac output and blood pressure (McArdle et al. 2010). Thus, such exercise may not be indicated for all patients with NAFLD.

Whole‐body vibration (WBV) has been recently introduced as a novel alternative. This exercise passively generates intense stimulation to produce dynamic changes in muscle fiber length through tonic vibration reflex. The technique uses rapid and repeated oscillations transmitted from a vibration device while the patient stands or sits on a platform. This form of exercise elicits strong stimulation of muscles with brief exposure to vibration (Cochrane 2010). Many previous studies have reported the efficacy of WBV in various fields of medicine and physiology (Cochrane 2010). WBV may be a suitable exercise approach for patients who have difficulties in engaging in general exercise due to its effectiveness and convenience of use.

In this study, we evaluated the effectiveness and usefulness of WBV in the management of patients with NAFLD, as an extension of a previous pilot study (Sumida et al. 2015). We conducted a 6‐month supervised WBV program and analyzed its effects on hepatic steatosis and stiffness, liver function tests, body adiposity, and metabolic parameters. We compared the outcomes with those of control patients with NAFLD who did not undergo the exercise regimen. In a separate analysis, to obtain specific information regarding the advantages of WBV, we measured markers of oxidative stress and inflammation, adipokines, morphological changes, and functional changes (ectopic fat deposition, muscle strength, and Biodex measurements), in patients with NAFLD who participated in the WBV program.

## Methods

### Study subjects

Forty‐five NAFLD patients who had difficulty engaging in regular exercise were recruited by physicians specializing in lifestyle‐related liver diseases in an outpatient department at the University of Tsukuba Hospital, Ibaraki prefecture, Japan, between January 2014 and December 2016. Specifically, patients were recruited whose NAFLD had been worsening, despite lifestyle counseling as part of a routine long‐term program offered by the hospital (mean >1 year). Each patient met the following inclusion criteria: NAFLD, according to the diagnostic guidelines of the Asia‐Pacific region (Chitturi et al. 2007); no signs or symptoms of heart disease; sedentary lifestyle (exercise frequency: ≤1 session per week and ≤30 min per session) over the past year; age ≥20 but ≤70; alcohol consumption <20 g per day; no other causes of liver disease; no suspicion of liver cirrhosis (Castera et al. 2008), assessed by elastography (<12.5 kPa); and with permission to exercise from their physician.

Of the recruited patients, 25 participated in the WBV program for 6 months as the WBV group (four men and 21 women; mean age: 54.2 years) and all completed it. In addition, another 20 patients participated in the study as the control (CON) group. This group received 12–24 sessions of lifestyle counseling regarding diet and physical activity, conducted by a registered dietitian and a physician specializing in nutrition, over the 6‐month period. This counseling is conducted as part of the routine management undertaken at the hospital. Three patients dropped out of the study from the CON group (one abandoned the study and two dropped out because of lack of time). Accordingly, we analyzed data from 17 patients with NAFLD (five men and 12 women; mean age: 48.4 years) in the CON group. All the measurements were made prior to and 5 days after the completion of the intervention. To ensure accuracy, we asked all the patients to avoid exercise during the previous day and to fast overnight for 12 h (water intake was permitted) before the measurements. All measurements were carried out during the morning at 10.

This study was approved by the Institutional Review Board of the University of Tsukuba Hospital and retrospectively registered with the University Hospital Medical Information Network Clinical Trials Registry (UMIN‐CTR ID no: R000034782, 19, December 2017). The programs were carried out in accordance with the principles of the 1975 Declaration of Helsinki, and all subjects signed informed consent documents prior to participation in this study.

### WBV program

For this study, we used a WBV program with its intensity and time modified from a previous pilot study (Oh et al. 2014): the strength was slightly increased, and the duration was reduced by half. The patients in the WBV group visited the rehabilitation division of Tsukuba University Hospital and underwent a WBV program designed to train the upper and lower body using the following three procedures: (1) Preparation: hamstring stretch, calf stretch, side stretch, and hip joint stretch (frequency: 30–35 Hz, amplitude: low, time: 30 sec, number: 1); (2) Strength and power: deep squat, wide stance squat, lunge, push up, triceps dip, crunch, front plank, and pelvic bridge (frequency: 30–35 Hz, amplitude: low, time: 30 sec, number: 1); (3) Massage: calf, hamstring, lower back, shoulder, and face (frequency: 40–50 Hz, amplitude: high, time: 30 sec, number: 1); using a Power Plate Pro‐6 (Badhoevendorp, Netherlands) and lasting a total of ~20 min (≥53 kcal, ≤76 kcal), without a rest interval after each movement, twice a week for 6 months.

The maximum pulse rate of the patients increased in proportion to the exercise intensity involved in each procedure versus their resting pulse rate (preparation session: +11 bpm, strength and power session: +45 bpm, massage session: +16 bpm) (OH1, Polar Electro Inc., Bethpage, NY). Their systolic (mean −6.2 mmHg) and diastolic (mean −1.6 mmHg) blood pressures were reduced during the WBV program (HEM‐770A, Omron Health Care, Kyoto, Japan).

**Figure 1:**
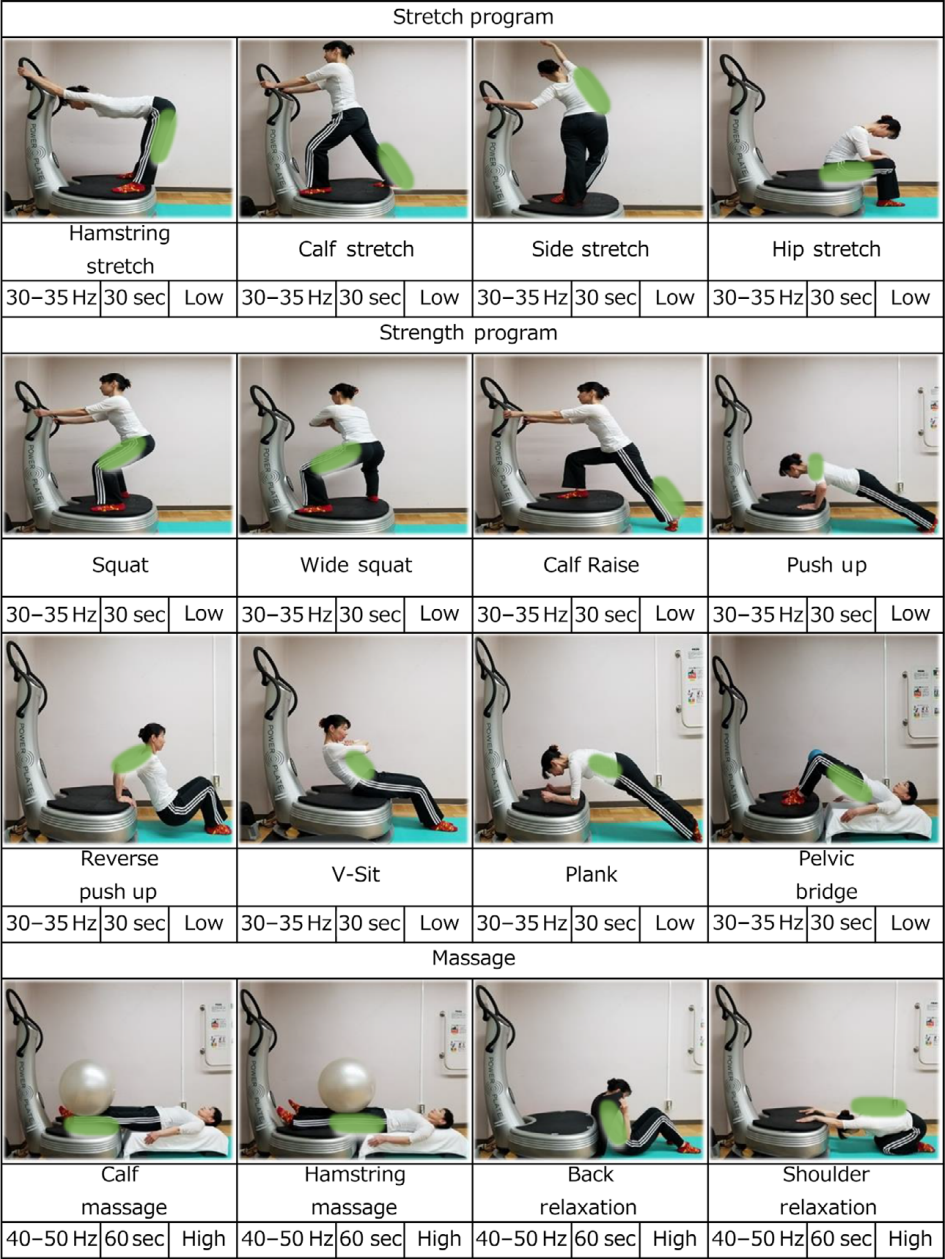
The program chart for whole‐body vibration exercise.

Figure 1 displays details of the WBV program. The procedures were carried out with the assistance of certified professional trainers and under the supervision of a study physician.

In our previous pilot study (Oh et al. 2014), we identified a number of problems related to the capability and willingness of patients to perform WBV exercise, and we modified the protocol to avoid these in the present study. Our staff has a great deal of experience of exercise intervention studies, and our WBV trainers have all been trained in the specific exercises involved and are highly specialized, with a lot of field experience. They assisted the patients on a one‐to‐one basis and corrected their positions to provide maximum stimulation to each of the appropriate muscles. For example, in the case of push‐ups, the knees were placed on the floor and the load of their body weight was placed on their arms, which leads to gradual achievement of the proper posture. In addition, the load encountered while undertaking reverse push‐ups was controlled by setting an appropriate angle for the elbow to be flexed, by adjusting the space between the hands and legs. The proper posture and stability of patients with muscular deficits was ensured during virtually all body movements by the trainer. Because patients with NAFLD often have an enormous variety of different symptoms, it is important for the purposes of treatment that they are assisted by a professional trainer. The subjects were asked not to participate in any lifestyle counseling, and to maintain their normal diet and exercise routines.

### Anthropometry and body composition

Height was measured with a wall‐mounted stadiometer (Muratec‐KDS, Kyoto, Japan). Body weight, fat mass, skeletal muscle mass, and abdominal visceral fat area were analyzed using an InBody720 (Biospace Japan, Tokyo, Japan), with the fasted patients being dressed in light clothing, after urinating, and after 10 min in a standing position. BMI was calculated (kg·m^−2^) using these data. We calculated a new surrogate marker for NAFLD, “skeletal muscle mass to visceral fat area ratio” (SV‐ratio) (Moon et al. 2013) in units of g·cm^−2^.

### Laboratory determinations

Blood was drawn from the right median cubital vein using a butterfly needle at baseline and at the sixth month while the patient was fasting. Serum and plasma samples were separated using low‐speed centrifugation (Tokyo, Japan). We measured triglyceride (TG), total cholesterol (TC), low‐density lipoprotein cholesterol (LDL), and free fatty acid (FFA) levels as follows: aspartate aminotransferase (AST), alanine aminotransferase (ALT), and gamma‐glutamyl transpeptidase (*γ*GT) using the Japan Society of Clinical Chemistry transferable method; Hyaluronan (HA) and plasma type IV collagen using the latex agglutination method; plasma insulin and ferritin levels using a chemiluminescent immunoassay method; and plasma glucose levels using the hexokinase‐G‐6‐PDH method. For the 25 subjects who completed the WBV program, we evaluated serum levels of thiobarbituric acid reactive substances (TBARS; Cayman, Ann Arbor, USA), tumor necrosis factor alpha (TNF‐*α*), interleukin 6 (IL6) and leptin (R&D Systems; Minneapolis, USA), M30 (Peviva AB, Bromma, Sweden), high‐sensitivity C‐reactive protein (hsCRP; Calbiotech, Spring Valley, USA), and adiponectin (Sekisui Medical, Tokyo, Japan) using commercial enzyme‐linked immunosorbent assay kits. Other markers included the homeostatic model assessment‐insulin resistance (HOMA‐IR), used to quantify insulin resistance (Matthews et al. 1985) and the NAFLD fibrosis score, used to measure degree of liver fibrosis (Angulo et al. 2007).

### Assessment of Fibroscan^®^ and magnetic resonance spectroscopy (^1H^MRS)

Hepatic stiffness was determined using Fibroscan^®^ (Echosens, Paris, France) with a 3.5 MHz standard probe. Details regarding the method have been published previously (Sandrin et al. 2003). Hepatic fat quantity was determined with a controlled attenuation parameter (CAP) designed to evaluate hepatic ultrasonic attenuation with a 3.5 MHz standard probe, using signals acquired by the Fibroscan. Detailed procedures have also been published previously (Sasso et al. 2010). Muscle area, intramyocellular lipids (IMCL), extramyocellular lipids (EMCL) in both quadriceps muscles, and intrahepatic lipids (IHL) were used to calculate the ratio of the intrahepatic fat signal to water signal (lipid·water^‐1^). These parameters were analyzed using a 3‐tesla magnetic resonance device (Achieva; Philips Electronics Japan, Tokyo, Japan) equipped with a 6‐channel Torso coil, as previously described (Sumida et al. 2014). The acquired data were analyzed with LCModel software (LA Systems, Tokyo, Japan).

### Muscle strength

Isometric knee flexion and extension strength was determined using a Biodex System III dynamometer (Biodex Medical Systems, Shirley, USA). The knee was extended to an angle of 60° in a seated position to produce maximal power of the quadriceps with close‐to‐optimum muscle lengths. Peak torque (Nm) consisting of three maximal efforts was determined as the maximal strength. Each isometric contraction was maintained for 3 seconds. The results were normalized to body weight (Kg) (Oh et al. 2015a,b).

### Statistical analysis

Statistical analysis was conducted using the Statistical Package for the Social Sciences (SPSS) for Windows, version 23.0 (IBM, Armonk, USA). Descriptive values were presented as mean ± standard error (SE) or standard deviation (SD), or log transformations for skewed variables. Analysis of categorical parameters was conducted using the chi‐squared test. For analysis of significant differences in baseline among the groups, the one‐way analysis of variance (ANOVA) test was used. To evaluate significant changes (at baseline and at 6 months) in clinical values within each group, we conducted paired *t* tests. Significant differences between groups (CON group vs. WBV group) were evaluated using either independent *t* tests test or analysis of covariance (ANCOVA). *P *< 0.05 was considered statistically significant.

## Results

A total of 42 patients with NAFLD participated, including 17 in the CON group and 25 in the WBV group. The attendance rate was 89.3% in the WBV group. Patients in the WBV group were further analyzed to determine the effects of WBV training on NAFLD pathophysiology, including measurements of intramyocellular and intrahepatic lipid deposition, muscle area and strength, adipokines, apoptosis, inflammation, and oxidative stress.

### Baseline characteristics

At baseline, there were no significant differences between groups in terms of age, alcohol consumption, and smoking. In addition, no measurements, including anthropometry, ectopic fat, hepatic stiffness, liver function test, insulin resistance, and lipid profile (with the exception of LDLC) were statistically different between the CON and WBV groups.

### Anthropometry

Table 1(a) displays anthropometry for each group during the study period. In the WBV group, comparing baseline with the 6‐month results, body weight decreased by 2.5%, BMI by 2.3%, fat mass by 6.8%, SV‐ratio increased by 7.9%, percentage fat mass decreased by 4.5%, and VAT area decreased by 6.2%. In the CON group, we observed significant changes in three parameters: fat mass increased by 2.4%, SV‐ratio decreased by 5.18%, percentage fat mass increased by 2.7%, and VAT area increased by 3.3% at 6 months. In the CON group, body weight was unchanged by the end of the study. When a comparison was made between the two groups, the magnitude of changes in all anthropometric parameters was greater in the WBV group compared with those of the CON group.

**Table 1:** The outcomes of anthropometry and body composition, metabolic parameters (Insulin Resistance and Lipid Profile) values in a total of 42 subjects with NAFLD

	CON				WBV				CON vs. WBV
	*n* = 17				*n* = 25				
	Pre	Post	Change	*P*	Pre	Post	Change	*P*	*P*
(a) Anthropometry and body composition									
BMI, kg m^−2^	29.2 ± 5.5	29.3 ± 5.4	+0.1	0.863	29.9 ± 6.9	29.2 ± 6.6	−0.7	0.008	0.010
Body weight, kg^−1^	78.4 ± 24.6	78.3 ± 24.4	−0.1	0.791	76.3 ± 16.2	74.4 ± 15.9	−1.9	0.005	0.009
Fat mass, kg^−1^	29.4 ± 12.0	30.2 ± 12.2	+0.7	0.026	30.9 ± 12.2	28.8 ± 11.7	−2.1	0.000	0.000
SVR	200.5 ± 64.3	190.1 ± 58.4	−10.4	0.001	197.0 ± 49.0	212.6 ± 55.1	+15.6	0.000	0.000
% Fat mass	37.0 ± 5.8	38.0 ± 6.0	+1.0	0.005	39.7 ± 8.1	37.9 ± 7.8	−1.8	0.000	0.000
VAT area, cm^2^	129.4 ± 31.3	133.7 ± 31.4	+4.3	0.003	130.7 ± 34.3	122.6 ± 34.1	−8.1	0.000	0.000
(b) Metabolic parameter									
_Log_FPG	2.044 ± 0.092	2.069 ± 0.122	+0.025	0.040	2.059 ± 0.130	2.040 ± 0.092	−0.019	0.205	0.039
_Log_FPI	1.066 ± 0.389	1.032 ± 0.428	−0.034	0.412	1.028 ± 0.270	0.976 ± 0.341	−0.052	0.356	0.795
_Log_HOMA−IR	0.514 ± 0.414	0.514 ± 0.453	0.000	0.994	0.480 ± 0.352	0.409 ± 0.401	−0.071	0.255	0.337
LDLC, mg dL^−1^ a	134.8 ± 32.3	135.1 ± 34.0	+0.3	0.972	114.8 ± 27.6	109.9 ± 25.6	−4.9	0.097	0.547
_Log_Triglycerides	2.162 ± 0.262	2.222 ± 0.262	+0.060	0.108	2.084 ± 0.198	2.040 ± 0.200	−0.044	0.150	0.030
_Log_FFA	1.781 ± 0.131	1.758 ± 0.178	−0.023	0.458	1.792 ± 0.134	1.706 ± 0.141	−0.110	0.001	0.025

Values are presented as the group means ± SD. ^a^ANCOVA with adjustments for respective baseline values were applied to compare changed values between groups. Abbreviations: CON, control group; WBV, whole‐body vibration; BMI, body mass index; SVR, skeletal muscle mass to visceral fat area ratio; VAT, visceral adipose tissue; FPG, fasting plasma glucose; FPI, fasting plasma insulin; HOMA‐IR, insulin resistance by homeostasis model; FFAs, LDLC, low‐density lipoprotein cholesterol; FFA, free fatty acids.

### Ectopic fat, hepatic stiffness, and liver function tests

**Figure 2:**
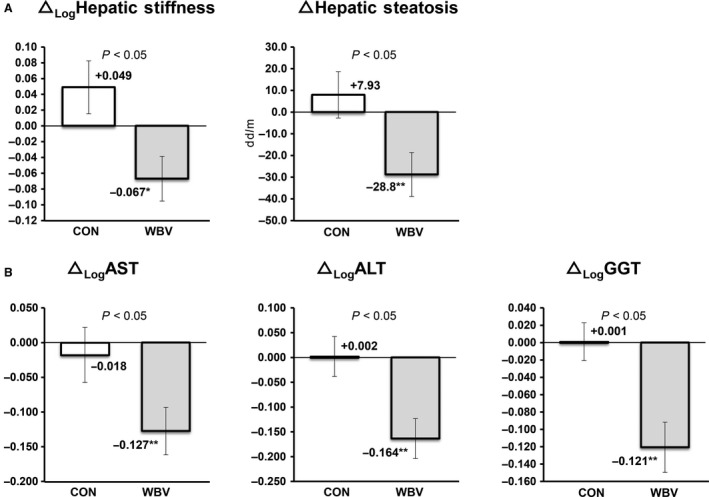
Changes in levels of hepatic steatosis and stiffness (A), liver function tests (B), from the baseline to the end point of 12 weeks in 25 subjects with NAFLD who participated in a 6‐month whole‐body vibration exercise program.

Figure 2 shows the results of hepatic fat and stiffness for the WBV group. Hepatic fat decreased by 10.0% at 6 months. In the CON group, hepatic fat increased by 2.9% (not significant). The WBV group showed decreases in hepatic stiffness of 8.3%. All liver function tests improved in the WBV group from the baseline to 6 months: AST decreased by 8.5%, ALT decreased by 10.6%, and *γ*GT decreased by 7.8%. There were no statistical differences in the CON group between the beginning and the end of the study. When a comparison was made between the CON and WBV groups, the magnitude of the decrease in levels of all parameters was greater in the WBV group (*P *< 0.05).

### Metabolic parameters

The results of changes in the insulin resistance and lipid profile are displayed in Table 1(b). From baseline to 6 months, FPG increased by 1.2% in the CON group, while no increase was observed in the WBV group. No other parameter showed a significant change in either the CON or the WBV group. In addition, metabolic parameters were not significantly different between the two groups.

On lipid profile analysis, FFA decreased by 16.8% in the WBV group. The other parameters did not significantly change in either the CON group or the WBV group over the course of the study. However, a comparison between the groups revealed that the magnitude of the decrease in triglycerides and FFA levels were greater in the WBV group than in the CON group (*P *< 0.01).

### Intramyocellular and intrahepatic lipid deposition

**Figure 3:**
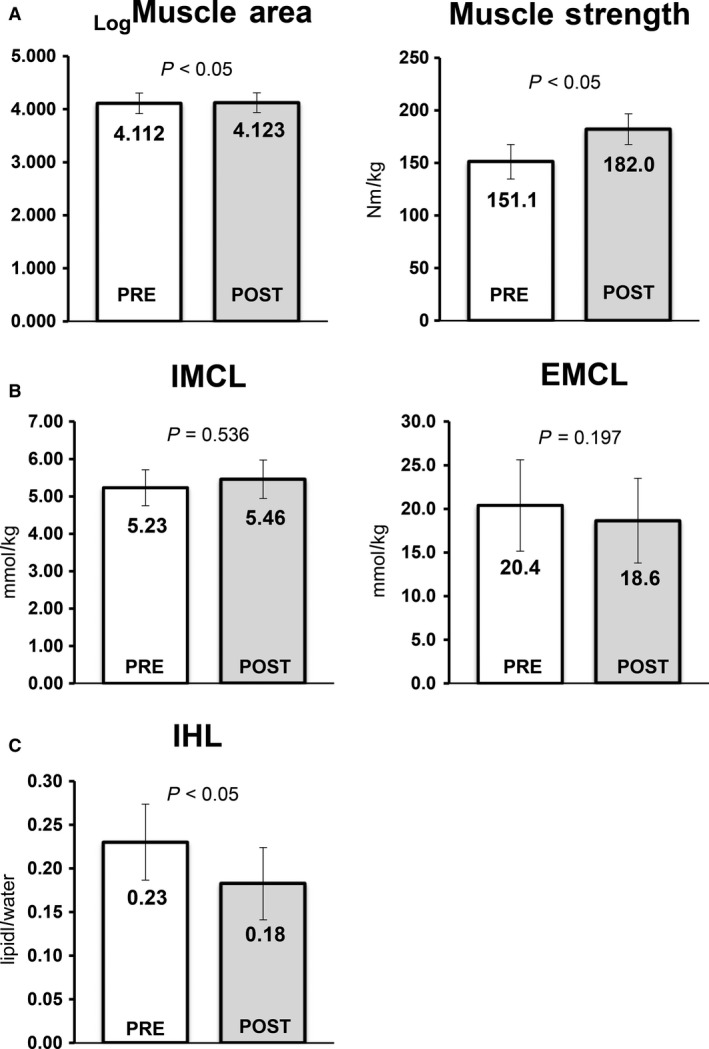
Changes in skeletal muscle area and strength in quadriceps (A), extra‐, intramyocellular lipids (B), and intrahepatic lipids (C) from baseline to 6 months in 25 subjects with NAFLD who participated in a 6‐month whole‐body vibration exercise program. Abbreviation: IMCL, intramyocellular lipids; IHL, intrahepatic lipids.

Figure 3 shows the results of changes in IMCL and EMCL (B) and IHL (C) assessed using ^1H^MRS for the WBV group during the intervention period. ^1H^MRS analysis revealed significant decreases IHL levels (21.7%) in the WBV group. However, IMCL levels in the quadriceps showed no significant changes.

### Muscle area and strength

Figure 3A shows the results of changes in muscle area assessed by ^1H^MRS analysis and muscle strength using Biodex in the quadriceps for the WBV group after 6 months.

In addition to a significant increase in skeletal muscle area of the quadriceps by 2.7%, muscle strength in the quadriceps increased by 18.4%.

### Adipokines, apoptosis, inflammation, and oxidative stress

**Figure 4:**
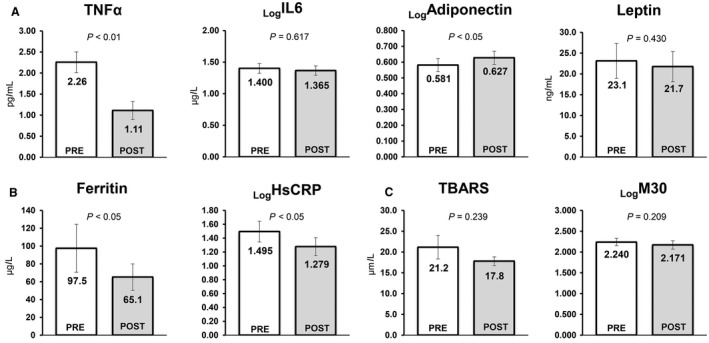
Changes in levels of adipokines (A), inflammation (B) and markers of apoptosis and oxidative stress (C), from baseline to 12 weeks in 25 subjects with NAFLD who participated in a 6‐month whole‐body vibration exercise program. Abbreviation: TNF‐*α*, tumor necrosis factor alpha; TBARS, thiobarbituric acid reactive substances; IL6, interleukin 6; hsCRP, high‐sensitivity CRP.

Serum markers of adipokines (TNF*α*, IL6, adiponectin, and leptin), oxidative stress (TBARS), hepatic inflammation (ferritin and HsCRP), and hepatocyte apoptosis (M30) were determined in the WBV group (Fig. 4). After the WBV program, significant changes were observed in three parameters: TNF*α* decreased by 50.8%, adiponectin increased by 7.9%, ferritin decreased by 18.0%, and HsCRP decreased by 14.5%. There were no significant changes in levels of IL6, leptin, TBARS, or M30.

## Discussion

The patients with NAFLD who underwent the WBV program for 6 months showed a modest decrease in body weight (Table 1a). Weight reduction is generally accepted to be the most effective therapy for NAFLD in clinical practice (Harrison and Day 2007). Many studies have suggested that an approximate 3–10% reduction in weight alters liver function tests, metabolic abnormalities, and the degree of hepatic steatosis (Keating and Adams 2016). In this study, a weight reduction of only 2.5% was observed in the WBV group. However, we noted significant improvements in muscle area and strength in the quadriceps muscle (Fig. 3) coupled with an increased SV‐ratio and decreased levels of whole‐body fat mass (Table 1a). Moreover, we observed considerable decreases in ectopic fat deposition, hepatic fat content (Fig. 2), IHL (Fig. 3C), VAT (Table 1a), FPG, and FFA (Table 1b). In the WBV group, there was improvement with respect to hepatic stiffness (Fig. 2), as well as the lowered levels of inflammatory markers (TNF*α*, adiponectin, ferritin, and hsCRP, Fig. 4). In addition, there were improvements in liver function tests, including ALT, AST, and *γ*GT (Fig. 2). Recently, it has been demonstrated that exercise meliorates NAFLD independent of detectable reductions in body weight. This is not to say that weight reduction is not very important in managing NAFLD. However, our results suggest that WBV may be practical and effective for patients that have difficulty engaging in regular exercise.

Skeletal muscle is the most abundant tissue of the human body, comprising 40–50% of total body mass. Skeletal muscle is not only the tool for mechanical movements but also plays a significant role in glucose and fatty acid metabolism regulating insulin sensitivity (Turcotte and Fisher 2008). Furthermore, skeletal muscle acts as a secretory organ for myokines (Pedersen 2013), which mediate crosstalk between various organs, including liver and adipose tissue. Bogaerts et al. (2007) and Machado et al. (2010) performed randomized controlled trials showing that WBV in elderly patients produces an explosive increase in muscle size and strength via strong eccentric overload generated by the vibration. This result compared favorably with a general exercise program (Stewart et al. 2014), consistent with our results. Although controversial, it has been reported that WBV alters steroid and growth hormones related to muscle hypertrophy, such as increases in testosterone, growth hormone, and insulin‐like growth factor 1, as well as decreases in cortisol (Domingos et al. 2015). Positive changes in these hormones induced by WBV may lead to significant improvement in muscle size and strength. Interestingly, the pathological conditions associated with NAFLD have been closely linked to testosterone (Kim et al. 2012), growth hormone (Takahashi et al. 2007), insulin‐like growth factor 1 (Sumida et al. 2015), and cortisol (Targher et al. 2006). WBV may be partially responsible for the activation of the myokine irisin, which transforms white fat cells into brown fat (Huh et al. 2014). The strong vibration of muscle WBV mimics the normal cold response, giving rise to increases in irisin levels (Huh et al. 2014). It was reported that increased irisin levels parallel increases in testosterone and insulin‐like growth factor 1(Huh et al. 2014), and is inversely related with degree of hepatic steatosis (Zhang et al. 2013). Therefore, the increased muscle size and strength stimulated by WBV might be a key factor in reversing pathology in NAFLD patients.

Excessive VAT deposition may be causally related to insulin resistance via the elaboration of excessive FFAs and abnormal adipokine balance. Koda et al. (2007) reported that visceral fat may be the most important risk factor for progression of NAFLD. We found that WBV improved levels of VAT as well as skeletal muscle in patients with NAFLD. WBV may lead to the improvement of systemic glucose and fatty acid metabolism, as well as that of adipokines. We suggest that improved levels of VAT (Despres and Lemieux 2006) and adiponectin (Kim et al. 2007) in our study may be associated with functional improvements in adipose tissue. These changes may reverse insulin resistance in adipose tissue, which would in turn inhibit the “lipotoxic” environment, and would lead to reduction in systemic levels of FFAs in patients with NAFLD in their daily life (Morigny et al. 2016). In addition, during WBV, circulating FFAs broken down in systemic adipose tissues and liver could be used to produce energy (Turcotte et al. 1992). Similarly, our observation of decreased levels of FPG may explain the observation of increased sensitivity to insulin for glucose uptake and continuous glycogen synthesis in active skeletal muscle (Robergs and Roberts 2000) during WBV. The decreased FPG associated with WBV is equivalent to that associated with other general exercise (Behboudi et al. 2011). A marked increased SV‐ratio suggested the importance of skeletal muscle and visceral fat for the hepatic aspects of NAFLD (Moon et al. 2013; Shida et al. 2017). Consequently, in daily life, beneficial ability to suppress lipolysis, and during WBV exercise, the availability of adipose tissue in the liver to produce energy that might otherwise contribute to hepatic steatosis.

Our previous study (Oh et al. 2017) and that of Kistler et al. (2011) found that only high‐intensity exercise improved inflammatory markers and hepatic stiffness. Therefore, the intensity of the exercise may be an important factor. Another point in favor of the superiority of WBV with respect to advanced fibrosis is the remarkable improvement in inflammatory markers achieved. The reductions in hepatic hsCRP and stiffness achieved in patients undergoing the low‐intensity WBV program suggest that this may be considered the best program for patients who have difficulty engaging in exercise. However, more specific and concrete measures, including liver biopsy and histology, are required to confirm its efficacy.

Our results may help begin to describe the molecular mechanisms mediating the effects of exercise on hepatic inflammation and fibrosis. First, in NAFLD patients, a great amount of iron accumulation in the liver has been observed. When combined with imbalanced adipokine secretion, this could stimulate development of inflammation and progression of liver fibrosis. Thus, the decreased level of serum ferritin resulting from 6‐month WBV exercise may contribute to the reduction of hepatic stiffness. Second, Savvidou et al. (2009) demonstrated that low serum adiponectin levels are a predictive factor of advanced fibrosis in the liver based on histologic parameters in biopsy‐proven NAFLD. Ding et al. (2005) found that adiponectin protects against hepatic advanced fibrosis via hepatic stellate cell apoptosis mediated by induction of caspase activation and inhibition of proliferation. Another possible explanation was suggested by Wigg et al. (2001), who reported high serum TNF*α* levels in patients with NASH. TNF*α* interferes with insulin signaling via accelerating hepatic steatosis and proinflammatory markers, contributing to progression of NASH (Crespo et al. 2001). Therefore, our study showed that the remarkable improvement of hepatic stiffness likely contributed to raised serum adiponectin levels and reduced serum TNF*α* levels as a result of WBV.

Ectopic fat in skeletal muscle and the liver has been shown to cause insulin resistance associated with NAFLD via impaired insulin signaling by diacylglycerol activation of protein kinase C50. We assessed pre–post‐WBV levels of IMCL and IHL using (1H) MRS. We found significant improvement of IHL, but not of IMCL levels. Although, many diabetes studies have demonstrated a stronger relationship between IMCL and insulin resistance in muscle, there continues to be a debate regarding the notion of the “athletic paradox” (Goodpaster et al. 2001) in the field of sports science. Briefly, despite the fact that trained athletes show elevated IMCL levels, they have noticeable insulin sensitivity and large oxidative capacity. This notion may apply to our observation of unchanged IMCL levels in the WBV group. WBV may allow preservation of local energy sources and increased oxidative capacity. In addition, WBV leads to considerable improvement in the degree of IHL in patients with NAFLD. Fabbrini et al. (2009) emphasized that IHL levels are among the most important indicators of metabolic abnormalities, including VAT levels. Increased IHL aggravates insulin resistance and increases hepatic very‐low‐density lipoprotein (VLDL) secretion rate. In addition, FFAs released by lipolysis of IHL increased hepatic VLDL production rate via an autocrine mechanism (Fabbrini et al. 2008). IHL was associated with hepatic glucose release, and correlated negatively with hepatic glycogen stores (Krssak et al. 2004). Therefore, noticeable reduction in IHL due WBV may be considered to be associated with improved liver function. In this regard, we observed improved liver function tests (AST, ALT, and *γ*GT) (Laker 1990) and amelioration of insulin resistance (Wallace et al. 2007).

The positive effects of general aerobic or resistance exercise are supported by evidence from many clinical trials on NAFLD pathophysiology. It appears that 4–12 weeks of aerobic (Johnson et al. 2009; Finucane et al. 2010; Van der Heijden et al. 2010; Keating et al. 2015b; Oh et al. 2017) or resistance (Hallsworth et al. 2011; Zelber‐Sagi et al. 2014; Oh et al. 2017) exercise led to a great improvement in the liver fat content, which is associated with metabolic comorbidities, compared with 24 weeks on our WBV program. These results may raise questions about the effectiveness of the WBV program. However, we recruited patients for this study whose NAFLD was worsening despite the lifestyle counseling that had been offered by the hospital for more than 1 year and most patients had difficulty engaging in general exercise because of physiological burdens. The patients’ attendance rate was about 90% for 6 months and they all completed the WBV program for 6 months without any injuries and medical problems. These study results may show a small improvement using WBV compared with general exercise. However, because WBV was considered to be a good alternative exercise method, patients, if they wished, could continue their exercise safely, conveniently, and efficiently in the hospital . However, it should be noted that there is currently little scientific evidence for the benefits of WBV and the optimal dose and modality for exercise therapy in NAFLD patients.

However, there were a few limitations to this study. First, it should be noted that the research protocol was not designed as a randomized controlled trial. The clinal trial was retrospectively registered because of an error. Overall, causality cannot be inferred from our results and there is a risk of bias. Second, we did not quantify daily energy intake and expenditure in this study. Although the subjects were asked not to participate in any lifestyle counseling and to maintain their normal diet and exercise routines. It would have aided the interpretation to quantify these outcomes. Finally, the lack of histological data prevented the evaluation of changes in the severity of NAFLD and fibrosis during the study.

In summary, we have shown that WBV provided benefit with respect to halting progression of NAFLD via reducing ectopic fat deposition in the liver and intra‐abdominal sites, as well as improvements in muscle morphology and function. In addition, WBV reduced hepatic stiffness and its related inflammatory markers that may contribute to enhanced innate immunity and dysregulation of signaling pathways in NAFLD. In short, these results demonstrate the usefulness of WBV as an exercise option for patients with NAFLD who have difficulty engaging in regular exercise.

## Conflict of Interest

The authors declare no competing financial interests.

## References

[phy214062-bib-0001] Angulo, P. 2002 Nonalcoholic fatty liver disease. N. Engl. J. Med. 346:1221–1231. 11961152 10.1056/NEJMra011775

[phy214062-bib-0002] Angulo, P. , J. M. Hui , G. Marchesini , E. Bugianesi , J. George , G. C. Farrell , et al. 2007 The NAFLD fibrosis score: a noninvasive system that identifies liver fibrosis in patients with NAFLD. Hepatology 45:846–854. 17393509 10.1002/hep.21496

[phy214062-bib-0003] Behboudi, L. , M.‐A. Azarbayjani , H. Aghaalinejad , and M. Salavati . 2011 Effects of aerobic exercise and whole body vibration on glycaemia control in type 2 diabetic males. Asian J. Sports Med. 2:83–90. 22375223 10.5812/asjsm.34789 PMC3289205

[phy214062-bib-0004] Bogaerts, A. , C. Delecluse , A. L. Claessens , W. Coudyzer , S. Boonen , and S. M. Verschueren . 2007 Impact of whole‐body vibration training versus fitness training on muscle strength and muscle mass in older men: a 1‐year randomized controlled trial. J. Gerontol. A Biol. Sci. Med. Sci. 62:630–635. 17595419 10.1093/gerona/62.6.630

[phy214062-bib-0005] Castera, L. , X. Forns , and A. Alberti . 2008 Non‐invasive evaluation of liver fibrosis using transient elastography. J. Hepatol. 48:835–847. 18334275 10.1016/j.jhep.2008.02.008

[phy214062-bib-0006] Chitturi, S. , G. C. Farrell , E. Hashimoto , T. Saibara , G. K. Lau , and J. D. Sollano . 2007 Non‐alcoholic fatty liver disease in the Asia‐Pacific region: definitions and overview of proposed guidelines. J. Gastroenterol. Hepatol. 22:778–787. 17565630 10.1111/j.1440-1746.2007.05001.x

[phy214062-bib-0007] Cochrane, D. J. 2010 ‘The effect of vibration exercise on aspects of muscle physiology and muscular performance’: a thesis submitted in partial fulfilment of the requirements for the degree of Doctor of Philosophy. Massey University, Palmerston North, New Zealand.

[phy214062-bib-0008] Crespo, J. , A. Cayon , P. Fernandez‐Gil , M. Hernandez‐Guerra , M. Mayorga , A. Dominguez‐Diez , et al. 2001 Gene expression of tumor necrosis factor alpha and TNF‐receptors, p55 and p75, in nonalcoholic steatohepatitis patients. Hepatology 34:1158–1163. 11732005 10.1053/jhep.2001.29628

[phy214062-bib-0009] Despres, J. P. , and I. Lemieux . 2006 Abdominal obesity and metabolic syndrome. Nature 444:881–887. 17167477 10.1038/nature05488

[phy214062-bib-0010] Ding, X. , N. K. Saxena , S. Lin , A. Xu , S. Srinivasan , and F. A. Anania . 2005 The roles of leptin and adiponectin: a novel paradigm in adipocytokine regulation of liver fibrosis and stellate cell biology. Am. J. Pathol. 166:1655–1669. 15920151 10.1016/S0002-9440(10)62476-5 PMC1602420

[phy214062-bib-0011] Domingos, L. L. , P. M. Giehl , D. N. Paiva , N. R. Asad , P. J. Marin , and M. Bernardo‐Filho . 2015 Alterations on the plasma concentration of hormonal and non hormonal biomarkers in human beings submitted to whole body vibration exercises. Sci. Res. Essays 10:287–297.

[phy214062-bib-0012] Fabbrini, E. , B. S. Mohammed , F. Magkos , K. M. Korenblat , B. W. Patterson , and S. Klein . 2008 Alterations in adipose tissue and hepatic lipid kinetics in obese men and women with nonalcoholic fatty liver disease. Gastroenterology 134:424–431. 18242210 10.1053/j.gastro.2007.11.038 PMC2705923

[phy214062-bib-0013] Fabbrini, E. , F. Magkos , B. S. Mohammed , T. Pietka , N. A. Abumrad , B. W. Patterson , et al. 2009 Intrahepatic fat, not visceral fat, is linked with metabolic complications of obesity. Proc. Natl Acad. Sci. USA 106:15430–15435. 19706383 10.1073/pnas.0904944106 PMC2741268

[phy214062-bib-0014] Finucane, F. M. , S. J. Sharp , L. R. Purslow , K. Horton , J. Horton , D. B. Savage , et al. 2010 The effects of aerobic exercise on metabolic risk, insulin sensitivity and intrahepatic lipid in healthy older people from the Hertfordshire Cohort Study: a randomised controlled trial. Diabetologia 53:624–631. 20052455 10.1007/s00125-009-1641-z

[phy214062-bib-0015] Goodpaster, B. H. , J. He , S. Watkins , and D. E. Kelley . 2001 Skeletal muscle lipid content and insulin resistance: evidence for a paradox in endurance‐trained athletes. J. Clin. Endocrinol. Metab. 86:5755–5761. 11739435 10.1210/jcem.86.12.8075

[phy214062-bib-0016] Hallsworth, K. , G. Fattakhova , K. G. Hollingsworth , C. Thoma , S. Moore , R. Taylor , et al. 2011 Resistance exercise reduces liver fat and its mediators in non‐alcoholic fatty liver disease independent of weight loss. Gut 60:1278–1283. 21708823 10.1136/gut.2011.242073 PMC3152868

[phy214062-bib-0017] Hardy, T. , Q. M. Anstee , and C. P. Day . 2015 Nonalcoholic fatty liver disease: new treatments. Curr. Opin. Gastroenterol. 31:175–183. 25774446 10.1097/MOG.0000000000000175 PMC4482455

[phy214062-bib-0018] Harrison, S. A. , and C. P. Day . 2007 Benefits of lifestyle modification in NAFLD. Gut 56:1760. 17911352 10.1136/gut.2006.112094 PMC2095707

[phy214062-bib-0019] Huh, J. Y. , V. Mougios , A. Skraparlis , A. Kabasakalis , and C. S. Mantzoros . 2014 Irisin in response to acute and chronic whole‐body vibration exercise in humans. Metabolism 63:918–921. 24814685 10.1016/j.metabol.2014.04.001

[phy214062-bib-0020] Johnson, N. A. , T. Sachinwalla , D. W. Walton , K. Smith , A. Armstrong , M. W. Thompson , et al. 2009 Aerobic exercise training reduces hepatic and visceral lipids in obese individuals without weight loss. Hepatology 50:1105–1112. 19637289 10.1002/hep.23129

[phy214062-bib-0021] Keating, S. E. , and L. A. Adams . 2016 Exercise in NAFLD: just do it. J. Hepatol. 65:671–673. 27392426 10.1016/j.jhep.2016.06.022

[phy214062-bib-0022] Keating, S. E. , J. George , and N. A. Johnson . 2015a The benefits of exercise for patients with non‐alcoholic fatty liver disease. Expert Rev. Gastroenterol. Hepatol. 9:1247–1250. 26289101 10.1586/17474124.2015.1075392

[phy214062-bib-0023] Keating, S. E. , D. A. Hackett , H. M. Parker , H. T. O'Connor , J. A. Gerofi , A. Sainsbury , et al. 2015b Effect of aerobic exercise training dose on liver fat and visceral adiposity. J. Hepatol. 63:174–182. 25863524 10.1016/j.jhep.2015.02.022

[phy214062-bib-0024] Kim, J.‐Y. , E. van de Wall , M. Laplante , A. Azzara , M. E. Trujillo , S. M. Hofmann , et al. 2007 Obesity‐associated improvements in metabolic profile through expansion of adipose tissue. J. Clin. Invest. 117:2621–2637. 17717599 10.1172/JCI31021 PMC1950456

[phy214062-bib-0025] Kim, S. , H. Kwon , J. H. Park , B. Cho , D. Kim , S. W. Oh , et al. 2012 A low level of serum total testosterone is independently associated with nonalcoholic fatty liver disease. BMC Gastroenterol. 12:69. 22691278 10.1186/1471-230X-12-69 PMC3406998

[phy214062-bib-0026] Kistler, K. D. , E. M. Brunt , J. M. Clark , A. M. Diehl , J. F. Sallis , and J. B. Schwimmer . 2011 Physical activity recommendations, exercise intensity, and histological severity of nonalcoholic fatty liver disease. Am. J. Gastroenterol. 106:460–468. 21206486 10.1038/ajg.2010.488 PMC3070294

[phy214062-bib-0027] Koda, M. , M. Kawakami , Y. Murawaki , and M. Senda . 2007 The impact of visceral fat in nonalcoholic fatty liver disease: cross‐sectional and longitudinal studies. J. Gastroenterol. 42:897–903. 18008034 10.1007/s00535-007-2107-z

[phy214062-bib-0028] Krssak, M. , A. Brehm , E. Bernroider , C. Anderwald , P. Nowotny , C. Dalla Man , et al. 2004 Alterations in postprandial hepatic glycogen metabolism in type 2 diabetes. Diabetes 53:3048–3056. 15561933 10.2337/diabetes.53.12.3048

[phy214062-bib-0029] Laker, M. F. 1990 Liver function tests. BMJ 301:250–251. 2202455 10.1136/bmj.301.6746.250 PMC1663456

[phy214062-bib-0030] Machado, A. , D. Garcia‐Lopez , J. Gonzalez‐Gallego , and N. Garatachea . 2010 Whole‐body vibration training increases muscle strength and mass in older women: a randomized‐controlled trial. Scand. J. Med. Sci. Sports 20:200–207. 19422657 10.1111/j.1600-0838.2009.00919.x

[phy214062-bib-0031] Matthews, D. R. , J. P. Hosker , A. S. Rudenski , B. A. Naylor , D. F. Treacher , and R. C. Turner . 1985 Homeostasis model assessment: insulin resistance and beta‐cell function from fasting plasma glucose and insulin concentrations in man. Diabetologia 28:412–419. 3899825 10.1007/BF00280883

[phy214062-bib-0032] McArdle, W. D. , F. I. Katch , and V. L. Katch . 2010 Exercise physiology: nutrition, energy, and human performance. Lippincott Williams & Wilkins, Philadelphia, PA.

[phy214062-bib-0033] Moon, J. S. , J. S. Yoon , K. C. Won , and H. W. Lee . 2013 The role of skeletal muscle in development of nonalcoholic fatty liver disease. Diabetes Metab. J. 37:278–285. 23991406 10.4093/dmj.2013.37.4.278 PMC3753493

[phy214062-bib-0034] Morigny, P. , M. Houssier , E. Mouisel , and D. Langin . 2016 Adipocyte lipolysis and insulin resistance. Biochimie 125:259–266. 26542285 10.1016/j.biochi.2015.10.024

[phy214062-bib-0035] Musso, G. , M. Cassader , F. Rosina , and R. Gambino . 2012 Impact of current treatments on liver disease, glucose metabolism and cardiovascular risk in non‐alcoholic fatty liver disease (NAFLD): a systematic review and meta‐analysis of randomised trials. Diabetologia 55:885–904. 22278337 10.1007/s00125-011-2446-4

[phy214062-bib-0036] Oh, S. , K. Tanaka , E. Warabi , and J. Shoda . 2013 Exercise reduces inflammation and oxidative stress in obesity‐related liver diseases. Med. Sci. Sports Exerc. 45:2214–2222. 23698242 10.1249/MSS.0b013e31829afc33

[phy214062-bib-0037] Oh, S. , T. Shida , A. Sawai , T. Maruyama , K. Eguchi , T. Isobe , et al. 2014 Acceleration training for managing nonalcoholic fatty liver disease: a pilot study. Ther. Clin. Risk Manag. 10:925–936. 25404857 10.2147/TCRM.S68322 PMC4230176

[phy214062-bib-0038] Oh, S. , T. Maruyama , K. Eguchi , T. Shida , E. Arai , T. Isobe , et al. 2015a Therapeutic effect of hybrid training of voluntary and electrical muscle contractions in middle‐aged obese women with nonalcoholic fatty liver disease: a pilot trial. Ther. Clin. Risk Manag. 11:371–380. 25767392 10.2147/TCRM.S75109 PMC4354454

[phy214062-bib-0039] Oh, S. , T. Shida , K. Yamagishi , K. Tanaka , R. So , T. Tsujimoto , et al. 2015b Moderate to vigorous physical activity volume is an important factor for managing nonalcoholic fatty liver disease: a retrospective study. Hepatology 61:1205–1215. 25271091 10.1002/hep.27544

[phy214062-bib-0040] Oh, S. , R. So , T. Shida , T. Matsuo , B. Kim , K. Akiyama , et al. 2017 High‐intensity aerobic exercise improves both hepatic fat content and stiffness in sedentary obese men with nonalcoholic fatty liver disease. Sci. Rep. 7:43029. 28223710 10.1038/srep43029 PMC5320441

[phy214062-bib-0041] Pedersen, B. K. 2013 Muscle as a secretory organ. Compr. Physiol. 3:1337–1362. 23897689 10.1002/cphy.c120033

[phy214062-bib-0042] Rector, R. S. , and J. P. Thyfault . 2011 Does physical inactivity cause nonalcoholic fatty liver disease? J. Appl. Physiol. 111:1828–1835. 21565984 10.1152/japplphysiol.00384.2011

[phy214062-bib-0043] Robergs, R. A. , and S. Roberts . 2000 Fundamental principles of exercise physiology: for fitness, performance, and health. McGraw‐Hill College, New York, NY.

[phy214062-bib-0044] Sandrin, L. , B. Fourquet , J. M. Hasquenoph , S. Yon , C. Fournier , F. Mal , et al. 2003 Transient elastography: a new noninvasive method for assessment of hepatic fibrosis. Ultrasound Med. Biol. 29:1705–1713. 14698338 10.1016/j.ultrasmedbio.2003.07.001

[phy214062-bib-0045] Sasaki, A. , H. Nitta , K. Otsuka , A. Umemura , S. Baba , T. Obuchi , et al. 2014 Bariatric surgery and non‐alcoholic fatty liver disease: current and potential future treatments. Front. Endocrinol. 5:164. 10.3389/fendo.2014.00164 PMC4209858 25386164

[phy214062-bib-0046] Sasso, M. , M. Beaugrand , V. de Ledinghen , C. Douvin , P. Marcellin , R. Poupon , et al. 2010 Controlled attenuation parameter (CAP): a novel VCTE guided ultrasonic attenuation measurement for the evaluation of hepatic steatosis: preliminary study and validation in a cohort of patients with chronic liver disease from various causes. Ultrasound Med. Biol. 36:1825–1835. 20870345 10.1016/j.ultrasmedbio.2010.07.005

[phy214062-bib-0047] Savvidou, S. , P. Hytiroglou , H. Orfanou‐Koumerkeridou , A. Panderis , P. Frantzoulis , and J. Goulis . 2009 Low serum adiponectin levels are predictive of advanced hepatic fibrosis in patients with NAFLD. J. Clin. Gastroenterol. 43:765–772. 19525862 10.1097/MCG.0b013e31819e9048

[phy214062-bib-0048] Shida, T. , K. Akiyama , S. Oh , A. Sawai , T. Isobe , Y. Okamoto , et al. 2017 Skeletal muscle mass to visceral fat area ratio is an important determinant affecting hepatic conditions of non‐alcoholic fatty liver disease. J. Gastroenterol. 53:535–547. 28791501 10.1007/s00535-017-1377-3

[phy214062-bib-0049] Spassiani, N. A. , and J. L. Kuk . 2008 Exercise and the fatty liver. Appl. Physiol. Nutr. Metab. 33:802–807. 18641726 10.1139/H08-059

[phy214062-bib-0050] Stewart, V. H. , D. H. Saunders , and C. A. Greig . 2014 Responsiveness of muscle size and strength to physical training in very elderly people: a systematic review. Scand. J. Med. Sci. Sports 24:e1–e10. 24151875 10.1111/sms.12123

[phy214062-bib-0051] Sumida, Y. , A. Nakajima , and Y. Itoh . 2014 Limitations of liver biopsy and non‐invasive diagnostic tests for the diagnosis of nonalcoholic fatty liver disease/nonalcoholic steatohepatitis. World J. Gastroenterol. 20:475–485. 24574716 10.3748/wjg.v20.i2.475 PMC3923022

[phy214062-bib-0052] Sumida, Y. , Y. Yonei , S. Tanaka , K. Mori , K. Kanemasa , S. Imai , et al. 2015 Lower levels of insulin‐like growth factor‐1 standard deviation score are associated with histological severity of non‐alcoholic fatty liver disease. Hepatol. Res. 45:771–781. 25163357 10.1111/hepr.12408

[phy214062-bib-0053] Takahashi, Y. , K. Iida , K. Takahashi , S. Yoshioka , H. Fukuoka , R. Takeno , et al. 2007 Growth hormone reverses nonalcoholic steatohepatitis in a patient with adult growth hormone deficiency. Gastroenterology 132:938–943. 17324404 10.1053/j.gastro.2006.12.024

[phy214062-bib-0054] Targher, G. , L. Bertolini , S. Rodella , G. Zoppini , L. Zenari , and G. Falezza . 2006 Associations between liver histology and cortisol secretion in subjects with nonalcoholic fatty liver disease. Clin. Endocrinol. 64:337–341. 10.1111/j.1365-2265.2006.02466.x 16487446

[phy214062-bib-0055] Turcotte, L. P. , and J. S. Fisher . 2008 Skeletal muscle insulin resistance: roles of fatty acid metabolism and exercise. Phys. Ther. 88:1279–1296. 18801860 10.2522/ptj.20080018 PMC2579902

[phy214062-bib-0056] Turcotte, L. P. , E. A. Richter , and B. Kiens . 1992 Increased plasma FFA uptake and oxidation during prolonged exercise in trained vs. untrained humans. Am. J. Physiol. 262:E791–E799. 1319676 10.1152/ajpendo.1992.262.6.E791

[phy214062-bib-0057] Van der Heijden, G. J. , Z. J. Wang , Z. D. Chu , P. J. Sauer , M. W. Haymond , L. M. Rodriguez , et al. 2010 A 12‐week aerobic exercise program reduces hepatic fat accumulation and insulin resistance in obese, Hispanic adolescents. Obesity 18:384–390. 19696755 10.1038/oby.2009.274

[phy214062-bib-0058] Wallace, T. M. , K. M. Utzschneider , J. Tong , D. B. Carr , S. Zraika , D. D. Bankson , et al. 2007 Relationship of liver enzymes to insulin sensitivity and intra‐abdominal fat. Diabetes Care 30:2673–2678. 17666458 10.2337/dc06-1758

[phy214062-bib-0059] Wigg, A. J. , I. C. Roberts‐Thomson , R. B. Dymock , P. J. McCarthy , R. H. Grose , and A. G. Cummins . 2001 The role of small intestinal bacterial overgrowth, intestinal permeability, endotoxaemia, and tumour necrosis factor alpha in the pathogenesis of non‐alcoholic steatohepatitis. Gut 48:206–211. 11156641 10.1136/gut.48.2.206 PMC1728215

[phy214062-bib-0060] Zelber‐Sagi, S. , A. Buch , H. Yeshua , N. Vaisman , M. Webb , G. Harari , et al. 2014 Effect of resistance training on non‐alcoholic fatty‐liver disease a randomized‐clinical trial. World J. Gastroenterol. 20:4382–4392. 24764677 10.3748/wjg.v20.i15.4382 PMC3989975

[phy214062-bib-0061] Zhang, H. J. , X. F. Zhang , Z. M. Ma , L. L. Pan , Z. Chen , H. W. Han , et al. 2013 Irisin is inversely associated with intrahepatic triglyceride contents in obese adults. J. Hepatol. 59:557–562. 23665283 10.1016/j.jhep.2013.04.030

